# Nicotinaldehyde [2,8-bis­(trifluoro­meth­yl)quinolin-4-yl]hydrazone monohydrate

**DOI:** 10.1107/S1600536810030862

**Published:** 2010-08-11

**Authors:** H. C. Devarajegowda, Suresh Babu Vepuri, M. VinduVahini, H. D. Kavitha, H. K. Arunkashi

**Affiliations:** a Department of Physics, Yuvaraja’s College (Constituent College), University of Mysore, Mysore 570 005, Karnataka, India; b Department of Pharmaceutical Chemistry, GITAM Institute of Pharmacy, GITAM University, Visakhapatnam 530045, Andhrapradesh, India; c Department of Physics, Sri D Devaraja Urs Govt. First Grade College, Hunsur 571 105, Mysore District, Karnataka, India; d Department of Physics, Govt. Science College, Hassan 573 201, Karnataka, India

## Abstract

In the title compound, C_17_H_10_F_6_N_4_·H_2_O, the pyridine ring is not coplanar with the quinoline ring system; the dihedral angle between the two planes is 21.3 (1)°. One of the trifluoro­methyl group is disordered over two orientations with occupancies of 0.70 (1) and 0.30 (1). The water mol­ecule is disordered over two positions with occupancies of 0.76 (1) and 0.24 (1). In the crystal, the water mol­ecule is linked to the main mol­ecule *via* N—H⋯O and C—H⋯O hydrogen bonds, and inversion-related pairs are linked *via* O—H⋯N hydrogen bonds. In addition, a weak π–π inter­action is observed between the pyridine ring and the pyridine ring of the quinoline unit, with a centroid–centroid distance of 3.650 (2) Å.

## Related literature

For general background to quinolines, see: Mao *et al.* (2009 ▶); Bermudez *et al.* (2004 ▶); Jayaprakash *et al.* (2006 ▶); Andries *et al.* (2005 ▶). For related structures, see: Al-eryani *et al.* (2010 ▶); Skörska *et al.* (2005 ▶).
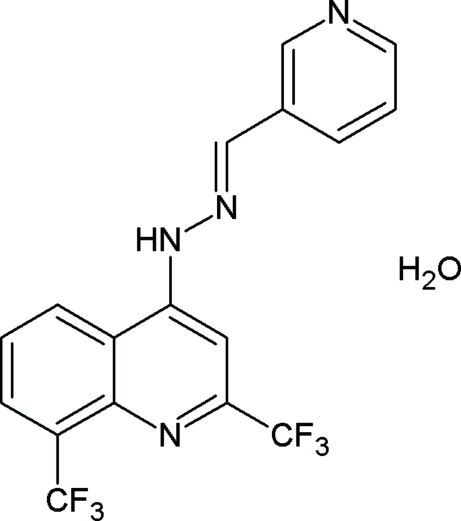


## Experimental

### 

#### Crystal data

C_17_H_10_F_6_N_4_·H_2_O
*M* *_r_* = 402.31
Monoclinic, 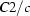

*a* = 21.103 (4) Å
*b* = 15.120 (3) Å
*c* = 12.537 (3) Å
β = 118.633 (3)°
*V* = 3511.1 (13) Å^3^
*Z* = 8
Mo *K*α radiation
μ = 0.14 mm^−1^
*T* = 295 K
0.22 × 0.15 × 0.12 mm

#### Data collection

Bruker SMART CCD area-detector diffractometer
Absorption correction: ψ scan (*SADABS*; Bruker, 2001 ▶) *T*_min_ = 0.975, *T*_max_ = 0.983
13267 measured reflections
3416 independent reflections
2309 reflections with *I* > 2σ(*I*)
*R*_int_ = 0.035

#### Refinement

*R*[*F* ^2^ > 2σ(*F* ^2^)] = 0.064
*wR*(*F* ^2^) = 0.169
*S* = 1.04
3416 reflections
301 parameters
54 restraints
H atoms treated by a mixture of independent and constrained refinement
Δρ_max_ = 0.26 e Å^−3^
Δρ_min_ = −0.25 e Å^−3^

### 

Data collection: *SMART* (Bruker, 2001 ▶); cell refinement: *SAINT* (Bruker, 2001 ▶); data reduction: *SAINT*; program(s) used to solve structure: *SHELXS97* (Sheldrick, 2008 ▶); program(s) used to refine structure: *SHELXL97* (Sheldrick, 2008 ▶); molecular graphics: *ORTEP-3* (Farrugia, 1997 ▶); software used to prepare material for publication: *SHELXL97* and *PLATON* (Spek, 2009 ▶).

## Supplementary Material

Crystal structure: contains datablocks I, global. DOI: 10.1107/S1600536810030862/ci5125sup1.cif

Structure factors: contains datablocks I. DOI: 10.1107/S1600536810030862/ci5125Isup2.hkl

Additional supplementary materials:  crystallographic information; 3D view; checkCIF report

## Figures and Tables

**Table 1:** Hydrogen-bond geometry (Å, °)

*D*—H⋯*A*	*D*—H	H⋯*A*	*D*⋯*A*	*D*—H⋯*A*
N2—H2*N*⋯O1*W*	0.88 (3)	2.04 (3)	2.914 (5)	169 (3)
O1*W*—H1*W*⋯N4^i^	0.83 (5)	2.06 (6)	2.855 (5)	163 (7)
C9—H9⋯O1*W*	0.93	2.42	3.331 (5)	167

Symmetry code: (i) 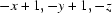.

**Fig. 1.**
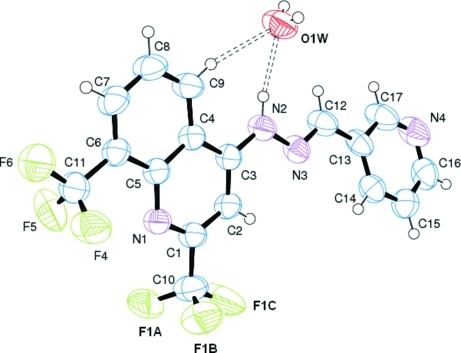
The asymmetric unit of the title compound. Displacement ellipsoids are drawn at the 50% probability level. Only the major disorder components are shown. Dashed lines indicate hydrogen bonds.

## supplementary crystallographic information

### Comment

2,8-Bis(trifluoromethyl)quinolin-4-yl]-(2-piperidyl)methanol (mefloquin) is a popular antimalarial drug. It also possesses important structural features required for antimicrobial activity (Mao *et al.* 2009; Bermudez *et al.* 2004; Jayaprakash *et al.* 2006). Quinoline is an essential structural unit found in mefloquin and recently developed antimycobacterial drugs (Andries *et al.* 2005). Thus, quinoline derivatives are good lead molecules to further develop drug candidates against mycobacterium tuberculosis and as antibacterial agents. On the basis of these observations we have synthesized few quinoline derivatives, in which a hydrazone group has been attached at the 4th position of the mefloquin ring system, expecting that these newly designed molecules would exhibit some antibacterial activity. The crystal structures of the mefloquine base and its salt complexes have been reported (Skörska *et al.* 2005). Earlier, we reported the crystal structure of 3,4-dimethoxybenzaldehyde [2,8-bis(trifluoromethyl)quinolin-4-yl]hydrazone (Al-eryani *et al.*, 2010). We report here the crystal structure of the title compound.

The asymmetric unit of the title compound contains one nicotinaldehyde [2,8-bis(trifluoromethyl)quinolin-4-yl]hydrazone molecule and one water molecule (Fig. 1). The dihedral angle [21.3 (1)°] between the quinoline ring system and the pyridine ring indicates that these two systems are non coplanar.

In the crystal structure, intermolecular O—H···N, N—H···O and C—H···O hydrogen bonds (Table 1) are observed. In addition, a weak π–π interaction is observed between N4/C13–C17 pyridine ring at (-x, y, 1/2-z) and N1/C1–C5 pyridine ring of quinoline at (x,y,z), with a centroid-centroid distance of 3.650 (3) Å.

### Experimental

A mixture of [2,8-bis(trifluoromethyl)quinolin-4-yl]hydrazine (10 mmol) and nicotinaldehyde (10 mmol) in glacial acetic acid (50 ml) was heated at reflux for 3 h. The reaction mixture was concentrated under reduced pressure, cooled, and the obtained solid hydrazone was filtered, washed with water and cold ethanol. The crude product was purified by column chromatography. Single crystals suitable for X-ray analysis were obtained by slow evaporation of an methanol-water solution at room temperature.

### Refinement

One of the trifluoromethyl group is disordered over two orientations with occupancies of 0.702 (8) and 0.298 (8). The C—F distances involving disordered F atoms were restrained to be equal and U^ij^ parameters of these atoms were restrained to an approximate isotropic behaviour. The water molecule is disordered over two positions with occupancies of 0.758 (13) and 0.242 (13); the H atoms of the major disorder component were located in a difference map and refined with O–H and H···H distances of 0.84 (2) and 1.35 (2) Å, respectively. Due to disorder of the oxygen atom in water molecule, all H atoms could not be  located. The N-bound H atom was located in a difference map and refined freely. The remaining H atoms were positioned at calculated positions [C–H = 0.93 Å] and refined using a riding model with *U*_iso_(H) = 1.2*U*_eq_(C).

### Crystal data

**Table:**

C_17_H_10_F_6_N_4_·H_2_O	*F*(000) = 1632
*M**_r_* = 402.31	*D*_x_ = 1.522 Mg m^−^^3^
Monoclinic, *C*2/*c*	Melting point: 486 K
Hall symbol: -C 2yc	Mo *K*α radiation, λ = 0.71073 Å
*a* = 21.103 (4) Å	Cell parameters from 3416 reflections
*b* = 15.120 (3) Å	θ = 1.7–26.0°
*c* = 12.537 (3) Å	µ = 0.14 mm^−^^1^
β = 118.633 (3)°	*T* = 295 K
*V* = 3511.1 (13) Å^3^	Plate, colourless
*Z* = 8	0.22 × 0.15 × 0.12 mm

### Data collection

**Table:**

Bruker SMART CCD area-detector diffractometer	3416 independent reflections
Radiation source: fine-focus sealed tube	2309 reflections with *I* > 2σ(*I*)
graphite	*R*_int_ = 0.035
ω and φ scans	θ_max_ = 26.0°, θ_min_ = 1.7°
Absorption correction: ψ scan (*SADABS*; Bruker, 2001)	*h* = −26→24
*T*_min_ = 0.975, *T*_max_ = 0.983	*k* = −18→18
13267 measured reflections	*l* = −15→15

### Refinement

**Table:**

Refinement on *F*^2^	Primary atom site location: structure-invariant direct methods
Least-squares matrix: full	Secondary atom site location: difference Fourier map
*R*[*F*^2^ > 2σ(*F*^2^)] = 0.064	Hydrogen site location: inferred from neighbouring sites
*wR*(*F*^2^) = 0.169	H atoms treated by a mixture of independent and constrained refinement
*S* = 1.04	*w* = 1/[σ^2^(*F*_o_^2^) + (0.0764*P*)^2^ + 2.423*P*] where *P* = (*F*_o_^2^ + 2*F*_c_^2^)/3
3416 reflections	(Δ/σ)_max_ = 0.001
301 parameters	Δρ_max_ = 0.26 e Å^−^^3^
54 restraints	Δρ_min_ = −0.25 e Å^−^^3^

### Special details

**Table:**

Geometry. All esds (except the esd in the dihedral angle between two l.s. planes) are estimated using the full covariance matrix. The cell esds are taken into account individually in the estimation of esds in distances, angles and torsion angles; correlations between esds in cell parameters are only used when they are defined by crystal symmetry. An approximate (isotropic) treatment of cell esds is used for estimating esds involving l.s. planes.
Refinement. Refinement of F^2^ against ALL reflections. The weighted R-factor wR and goodness of fit S are based on F^2^, conventional R-factors R are based on F, with F set to zero for negative F^2^. The threshold expression of F^2^ > 2sigma(F^2^) is used only for calculating R-factors(gt) etc. and is not relevant to the choice of reflections for refinement. R-factors based on F^2^ are statistically about twice as large as those based on F, and R- factors based on ALL data will be even larger.

### Fractional atomic coordinates and isotropic or equivalent isotropic displacement parameters (Å^2^)

**Table:**

	*x*	*y*	*z*	*U*_iso_*/*U*_eq_	Occ. (<1)
F1A	0.5904 (2)	−0.0211 (2)	0.5513 (4)	0.0998 (17)	0.702 (8)
F2A	0.6589 (4)	−0.0445 (5)	0.4831 (7)	0.136 (3)	0.702 (8)
F3A	0.5538 (5)	−0.0032 (4)	0.3634 (5)	0.175 (4)	0.702 (8)
F1B	0.6012 (6)	−0.0127 (6)	0.3579 (8)	0.091 (3)	0.298 (8)
F2B	0.5395 (6)	0.0067 (8)	0.4374 (17)	0.130 (5)	0.298 (8)
F3B	0.6570 (7)	−0.0477 (7)	0.5238 (10)	0.110 (6)	0.298 (8)
F4	0.80847 (12)	0.08843 (14)	0.83893 (19)	0.0933 (7)	
F5	0.71855 (14)	0.12394 (18)	0.8610 (2)	0.1087 (8)	
F6	0.81994 (15)	0.18889 (16)	0.96395 (17)	0.1198 (10)	
N1	0.68113 (12)	0.11985 (15)	0.6073 (2)	0.0554 (6)	
N2	0.61107 (14)	0.31173 (19)	0.3332 (2)	0.0623 (7)	
N3	0.56960 (13)	0.28345 (17)	0.2162 (2)	0.0607 (7)	
N4	0.42311 (17)	0.3601 (2)	−0.1996 (3)	0.0905 (10)	
C1	0.63497 (15)	0.10370 (18)	0.4929 (2)	0.0558 (7)	
C2	0.60955 (15)	0.16393 (18)	0.3984 (2)	0.0550 (7)	
H2	0.5766	0.1468	0.3198	0.066*	
C3	0.63410 (14)	0.24976 (18)	0.4233 (2)	0.0509 (7)	
C4	0.68355 (14)	0.27268 (18)	0.5461 (2)	0.0505 (7)	
C5	0.70505 (14)	0.20455 (17)	0.6345 (2)	0.0499 (7)	
C6	0.75344 (16)	0.22617 (19)	0.7576 (2)	0.0581 (8)	
C7	0.77952 (18)	0.3094 (2)	0.7890 (3)	0.0692 (9)	
H7	0.8118	0.3222	0.8698	0.083*	
C8	0.75865 (18)	0.3761 (2)	0.7019 (3)	0.0729 (9)	
H8	0.7769	0.4330	0.7248	0.087*	
C9	0.71174 (17)	0.35831 (19)	0.5837 (3)	0.0641 (8)	
H9	0.6980	0.4035	0.5265	0.077*	
C10	0.6071 (2)	0.0102 (2)	0.4681 (3)	0.0809 (11)	
C11	0.7746 (2)	0.1574 (2)	0.8540 (3)	0.0750 (10)	
C12	0.54235 (17)	0.3419 (2)	0.1340 (3)	0.0699 (9)	
H12	0.5489	0.4015	0.1546	0.084*	
C13	0.50072 (17)	0.3156 (2)	0.0070 (3)	0.0639 (8)	
C14	0.49612 (17)	0.2291 (2)	−0.0322 (3)	0.0662 (9)	
H14	0.5209	0.1843	0.0232	0.079*	
C15	0.45503 (19)	0.2098 (2)	−0.1525 (3)	0.0755 (10)	
H15	0.4508	0.1519	−0.1798	0.091*	
C16	0.4200 (2)	0.2770 (3)	−0.2323 (3)	0.0813 (11)	
H16	0.3926	0.2631	−0.3142	0.098*	
C17	0.4624 (2)	0.3780 (2)	−0.0816 (3)	0.0893 (12)	
H17	0.4642	0.4364	−0.0570	0.107*	
O1W	0.6581 (3)	0.4946 (2)	0.3473 (4)	0.088 (2)	0.758 (13)
H1W	0.631 (3)	0.537 (3)	0.315 (6)	0.132*	0.758 (13)
H2W	0.695 (2)	0.498 (4)	0.341 (7)	0.132*	0.758 (13)
O2W	0.6032 (15)	0.5042 (8)	0.3654 (12)	0.119 (10)	0.242 (13)
H2N	0.6237 (16)	0.368 (2)	0.345 (3)	0.065 (9)*	

### Atomic displacement parameters (Å^2^)

**Table:**

	*U*^11^	*U*^22^	*U*^33^	*U*^12^	*U*^13^	*U*^23^
F1A	0.121 (3)	0.070 (2)	0.112 (3)	−0.0409 (19)	0.059 (3)	−0.0055 (18)
F2A	0.200 (6)	0.079 (4)	0.169 (7)	0.015 (3)	0.120 (5)	−0.009 (4)
F3A	0.186 (6)	0.087 (3)	0.093 (3)	−0.068 (4)	−0.062 (3)	0.022 (3)
F1B	0.098 (6)	0.065 (4)	0.089 (6)	−0.019 (5)	0.027 (5)	−0.040 (4)
F2B	0.117 (8)	0.105 (7)	0.198 (12)	−0.053 (5)	0.099 (8)	−0.042 (7)
F3B	0.150 (9)	0.039 (4)	0.060 (5)	−0.002 (5)	−0.014 (5)	0.019 (4)
F4	0.0962 (15)	0.0700 (13)	0.0804 (14)	0.0121 (11)	0.0155 (12)	0.0131 (10)
F5	0.1159 (19)	0.126 (2)	0.0824 (15)	−0.0115 (15)	0.0462 (14)	0.0322 (13)
F6	0.156 (2)	0.0963 (16)	0.0446 (11)	−0.0156 (14)	−0.0024 (12)	0.0008 (10)
N1	0.0587 (14)	0.0482 (13)	0.0457 (13)	−0.0017 (11)	0.0141 (11)	0.0017 (10)
N2	0.0664 (17)	0.0551 (16)	0.0467 (13)	−0.0082 (12)	0.0122 (12)	0.0045 (11)
N3	0.0571 (15)	0.0648 (15)	0.0470 (13)	−0.0053 (12)	0.0144 (12)	0.0066 (11)
N4	0.098 (2)	0.087 (2)	0.0532 (16)	−0.0108 (17)	0.0094 (16)	0.0226 (16)
C1	0.0562 (17)	0.0488 (16)	0.0488 (15)	0.0003 (13)	0.0142 (13)	−0.0010 (12)
C2	0.0518 (16)	0.0546 (17)	0.0428 (14)	−0.0016 (13)	0.0099 (12)	−0.0031 (12)
C3	0.0465 (15)	0.0529 (16)	0.0458 (15)	0.0019 (12)	0.0162 (12)	0.0028 (12)
C4	0.0456 (15)	0.0509 (16)	0.0490 (15)	−0.0003 (12)	0.0178 (13)	−0.0010 (12)
C5	0.0463 (15)	0.0503 (16)	0.0455 (14)	0.0014 (12)	0.0158 (12)	0.0003 (12)
C6	0.0588 (18)	0.0577 (17)	0.0464 (15)	−0.0016 (14)	0.0160 (13)	−0.0028 (13)
C7	0.071 (2)	0.065 (2)	0.0496 (16)	−0.0067 (16)	0.0111 (15)	−0.0099 (15)
C8	0.076 (2)	0.0534 (18)	0.064 (2)	−0.0126 (16)	0.0141 (17)	−0.0098 (15)
C9	0.0663 (19)	0.0486 (17)	0.0614 (18)	−0.0040 (14)	0.0177 (15)	0.0032 (13)
C10	0.093 (3)	0.053 (2)	0.063 (2)	0.0027 (19)	0.010 (2)	−0.0039 (17)
C11	0.086 (3)	0.068 (2)	0.0471 (18)	−0.0073 (19)	0.0134 (17)	0.0009 (15)
C12	0.071 (2)	0.0597 (19)	0.0548 (18)	−0.0084 (15)	0.0108 (16)	0.0102 (14)
C13	0.0595 (18)	0.066 (2)	0.0538 (17)	−0.0075 (14)	0.0174 (15)	0.0150 (14)
C14	0.065 (2)	0.069 (2)	0.0581 (18)	0.0049 (16)	0.0240 (16)	0.0125 (15)
C15	0.082 (2)	0.080 (2)	0.059 (2)	0.0000 (19)	0.0292 (18)	−0.0011 (17)
C16	0.080 (2)	0.100 (3)	0.0503 (18)	−0.007 (2)	0.0203 (17)	0.0073 (19)
C17	0.098 (3)	0.070 (2)	0.062 (2)	−0.014 (2)	0.0081 (19)	0.0199 (17)
O1W	0.102 (4)	0.064 (2)	0.081 (2)	−0.010 (2)	0.029 (2)	0.0169 (17)
O2W	0.18 (2)	0.062 (7)	0.093 (9)	0.001 (8)	0.047 (10)	0.014 (6)

### Geometric parameters (Å, °)

**Table:**

F1A—C10	1.337 (5)	C4—C9	1.408 (4)
F2A—C10	1.312 (6)	C4—C5	1.419 (4)
F3A—C10	1.269 (5)	C5—C6	1.422 (4)
F1B—C10	1.370 (7)	C6—C7	1.354 (4)
F2B—C10	1.288 (8)	C6—C11	1.491 (4)
F3B—C10	1.288 (8)	C7—C8	1.394 (5)
F4—C11	1.328 (4)	C7—H7	0.93
F5—C11	1.328 (4)	C8—C9	1.357 (4)
F6—C11	1.334 (4)	C8—H8	0.93
N1—C1	1.314 (3)	C9—H9	0.93
N1—C5	1.359 (3)	C12—C13	1.457 (4)
N2—C3	1.365 (4)	C12—H12	0.93
N2—N3	1.367 (3)	C13—C14	1.384 (5)
N2—H2N	0.88 (3)	C13—C17	1.385 (4)
N3—C12	1.268 (4)	C14—C15	1.363 (4)
N4—C16	1.314 (5)	C14—H14	0.93
N4—C17	1.332 (4)	C15—C16	1.368 (5)
C1—C2	1.383 (4)	C15—H15	0.93
C1—C10	1.505 (4)	C16—H16	0.93
C2—C3	1.377 (4)	C17—H17	0.93
C2—H2	0.93	O1W—H1W	0.83 (2)
C3—C4	1.429 (4)	O1W—H2W	0.81 (2)
			
C1—N1—C5	116.2 (2)	F2B—C10—F1A	63.3 (8)
C3—N2—N3	117.6 (3)	F3B—C10—F1A	77.7 (8)
C3—N2—H2N	124 (2)	F2A—C10—F1A	100.2 (4)
N3—N2—H2N	118 (2)	F3A—C10—F1B	46.4 (5)
C12—N3—N2	117.5 (3)	F2B—C10—F1B	97.8 (8)
C16—N4—C17	116.9 (3)	F3B—C10—F1B	91.8 (8)
N1—C1—C2	126.4 (3)	F2A—C10—F1B	69.6 (6)
N1—C1—C10	114.4 (2)	F1A—C10—F1B	140.0 (5)
C2—C1—C10	119.2 (3)	F3A—C10—C1	115.4 (3)
C3—C2—C1	118.5 (2)	F2B—C10—C1	111.6 (6)
C3—C2—H2	120.7	F3B—C10—C1	112.9 (7)
C1—C2—H2	120.7	F2A—C10—C1	110.3 (5)
N2—C3—C2	120.9 (3)	F1A—C10—C1	113.2 (3)
N2—C3—C4	120.8 (3)	F1B—C10—C1	106.5 (5)
C2—C3—C4	118.3 (2)	F4—C11—F5	105.5 (3)
C9—C4—C5	118.7 (2)	F4—C11—F6	105.0 (3)
C9—C4—C3	123.8 (3)	F5—C11—F6	106.7 (3)
C5—C4—C3	117.5 (2)	F4—C11—C6	113.9 (3)
N1—C5—C4	123.2 (2)	F5—C11—C6	112.9 (3)
N1—C5—C6	118.5 (2)	F6—C11—C6	112.1 (3)
C4—C5—C6	118.4 (3)	N3—C12—C13	120.0 (3)
C7—C6—C5	120.7 (3)	N3—C12—H12	120.0
C7—C6—C11	119.3 (3)	C13—C12—H12	120.0
C5—C6—C11	120.0 (3)	C14—C13—C17	116.4 (3)
C6—C7—C8	120.8 (3)	C14—C13—C12	123.3 (3)
C6—C7—H7	119.6	C17—C13—C12	120.3 (3)
C8—C7—H7	119.6	C15—C14—C13	119.6 (3)
C9—C8—C7	120.2 (3)	C15—C14—H14	120.2
C9—C8—H8	119.9	C13—C14—H14	120.2
C7—C8—H8	119.9	C14—C15—C16	119.1 (4)
C8—C9—C4	121.2 (3)	C14—C15—H15	120.5
C8—C9—H9	119.4	C16—C15—H15	120.5
C4—C9—H9	119.4	N4—C16—C15	123.6 (3)
F3A—C10—F2B	51.7 (6)	N4—C16—H16	118.2
F3A—C10—F3B	122.8 (7)	C15—C16—H16	118.2
F2B—C10—F3B	129.4 (10)	N4—C17—C13	124.5 (4)
F3A—C10—F2A	108.2 (6)	N4—C17—H17	117.8
F2B—C10—F2A	138.2 (7)	C13—C17—H17	117.8
F3B—C10—F2A	23.7 (8)	H1W—O1W—H2W	112 (4)
F3A—C10—F1A	108.5 (6)		
			
C3—N2—N3—C12	173.2 (3)	N1—C1—C10—F3A	168.6 (8)
C5—N1—C1—C2	1.0 (5)	C2—C1—C10—F3A	−9.6 (9)
C5—N1—C1—C10	−177.1 (3)	N1—C1—C10—F2B	112.0 (9)
N1—C1—C2—C3	0.3 (5)	C2—C1—C10—F2B	−66.2 (10)
C10—C1—C2—C3	178.2 (3)	N1—C1—C10—F3B	−43.1 (9)
N3—N2—C3—C2	−7.4 (4)	C2—C1—C10—F3B	138.7 (8)
N3—N2—C3—C4	173.6 (3)	N1—C1—C10—F2A	−68.5 (5)
C1—C2—C3—N2	179.6 (3)	C2—C1—C10—F2A	113.3 (5)
C1—C2—C3—C4	−1.3 (4)	N1—C1—C10—F1A	42.9 (4)
N2—C3—C4—C9	0.1 (5)	C2—C1—C10—F1A	−135.3 (4)
C2—C3—C4—C9	−179.0 (3)	N1—C1—C10—F1B	−142.3 (6)
N2—C3—C4—C5	−179.9 (3)	C2—C1—C10—F1B	39.5 (7)
C2—C3—C4—C5	1.0 (4)	C7—C6—C11—F4	−120.2 (3)
C1—N1—C5—C4	−1.3 (4)	C5—C6—C11—F4	61.1 (4)
C1—N1—C5—C6	178.2 (3)	C7—C6—C11—F5	119.5 (4)
C9—C4—C5—N1	−179.7 (3)	C5—C6—C11—F5	−59.1 (4)
C3—C4—C5—N1	0.3 (4)	C7—C6—C11—F6	−1.1 (5)
C9—C4—C5—C6	0.8 (4)	C5—C6—C11—F6	−179.7 (3)
C3—C4—C5—C6	−179.1 (3)	N2—N3—C12—C13	176.9 (3)
N1—C5—C6—C7	179.1 (3)	N3—C12—C13—C14	−8.3 (5)
C4—C5—C6—C7	−1.4 (5)	N3—C12—C13—C17	170.9 (4)
N1—C5—C6—C11	−2.3 (4)	C17—C13—C14—C15	−0.3 (5)
C4—C5—C6—C11	177.2 (3)	C12—C13—C14—C15	179.0 (3)
C5—C6—C7—C8	1.1 (5)	C13—C14—C15—C16	1.0 (5)
C11—C6—C7—C8	−177.5 (3)	C17—N4—C16—C15	−0.5 (6)
C6—C7—C8—C9	−0.1 (6)	C14—C15—C16—N4	−0.6 (6)
C7—C8—C9—C4	−0.5 (5)	C16—N4—C17—C13	1.3 (6)
C5—C4—C9—C8	0.1 (5)	C14—C13—C17—N4	−0.9 (6)
C3—C4—C9—C8	−180.0 (3)	C12—C13—C17—N4	179.8 (4)

### Hydrogen-bond geometry (Å, °)

**Table:**

*D*—H···*A*	*D*—H	H···*A*	*D*···*A*	*D*—H···*A*
N2—H2N···O1W	0.88 (3)	2.04 (3)	2.914 (5)	169 (3)
O1W—H1W···N4^i^	0.83 (5)	2.06 (6)	2.855 (5)	163 (7)
C9—H9···O1W	0.93	2.42	3.331 (5)	167

Symmetry codes:  (i) −*x*+1, −*y*+1, −*z*.
